# Family practice nurses supporting self-management in older patients with mild osteoarthritis: a randomized trial

**DOI:** 10.1186/1471-2296-9-7

**Published:** 2008-01-28

**Authors:** Raymond Wetzels, Chris van Weel, Richard Grol, Michel Wensing

**Affiliations:** 1 Centre for Quality of Care Research (WOK), Radboud University Nijmegen Medical Centre, Nijmegen, The Netherlands; 2 Department of Family Practice, Radboud University Nijmegen Medical Centre, Nijmegen, The Netherlands

## Abstract

**Background:**

Supporting self-management intends to improve life-style, which is beneficial for patients with mild osteoarthritis (OA). We evaluated a nurse-based intervention on older OA patients' self-management with the aim to assess its effects on mobility and functioning.

**Methods:**

Randomized controlled trial of patients (≥ 65 years) with mild hip or knee OA from nine family practices in the Netherlands. Intervention consisted of supporting patients' self-management of OA symptoms using a practice-based nurse. Outcome measures were patients' mobility, using the Timed Up and Go test (TUG), and patient reported functioning, using an arthritis specific scale (Dutch AIMS2 SF).

**Results:**

Fifty-one patients were randomized to the intervention group and 53 to the control group. Patient-reported functioning improved on four scales in the intervention group compared to one scale in the control group. However, this result was not significant. Mobility improved in both groups, without a significant difference between the two groups. There were no differences between the groups regarding consultations with family physicians or physiotherapists, or medication use.

**Conclusion:**

A nurse-based intervention on older OA patients' self-management did not improve self-reported functioning, mobility or patients' use of health care resources.

## Background

In our aging population osteoarthritis (OA) is a highly prevalent chronic disease, which has a high impact on burden of disease, quality of life, and use of healthcare. Worldwide estimates are that 10% of men and 18% of women aged 60 years have symptomatic OA [1]. In early stages clinical management of OA is targeted at improving patients' self-management [2-5], losing weight [6], physical exercise [7-11] and adequate use of analgesics. But, medicalization of OA should be avoided. Patients' self-management may improve their life-style and therefore health outcomes, analogue to diabetic patients [12]. Healthcare systems face the challenge to enhance self-management in OA patients on a sufficiently large scale so that all patients are actually reached and helped. Barriers may be that improving patients' life-style often requires substantial investment of both patients' and health professionals' time, as many education programmes require a large number of sessions [13]. And, the health behaviors in older patients tend to be reserved, as they attribute many complaints towards getting older, and consequently arthritis symptoms are underreported [14]. Involving practice-based nurses in the management of OA ensures that this care is delivered closely to the patient. A recent review showed that substituting physicians for appropriately trained nurses could produce as highly quality care as primary care doctors and achieves as good outcomes for patients [15]. The availability of skilled nurses is limited and nursing time invested in any intervention needs to be examined critically. Therefore we wondered whether a single individual session with a trained nurse, which was focused on supporting patients' self-management, would be effective in OA patients. On the basis of previous research on changing life style behavior by family physicians (FPs), we expected a small but relevant change in patient behavior [16]. The aim of this proof of principle study was to evaluate the clinical effectiveness of a single session nurse-based intervention for enhancing self-management in older patients with mild OA.

## Methods

### Study Population

This study was a patient randomized controlled trial, which was performed between April 2004 and January 2005. This trial has not been registered beforehand in a publicly accessible trial registry as it was performed before prospective registering of these trials became obliged. The ethical committee of the Radboud University Medical Centre Nijmegen gave approval for the study.

The trial was based on the practice populations of seventeen FPs from nine urban non-academic practices in the Eastern region of the Netherlands. Patients were eligible if they were aged 65 or older and had been clinically diagnosed with OA of the hip or knee. The OA diagnosis needed to be registered in patient's practice medical history record as free text or as ICPC-code L89 (OA of the knee) or L90 (OA of the hip). Patients were excluded if they had undergone a hip or knee replacement operation, or had been referred for it or when their GP thought they were not suitable for participating (for example because of severe psychosocial circumstances, or a terminal disease). No further classification of degree of OA was made. An informed consent letter was sent by the GP and patients were included after they had replied positively.

### Randomization

An independent statistician made randomization lists in advance for each practice. To ensure similar numbers of patients from different practices in each group, block-randomization (blocks of two) was used. These randomization lists were represented in nine different spreadsheets. Every patient who entered the study was given a number that represented the order of entrance in the study for that practice. Subsequently, the number of entrance per practice in the spreadsheet was used to randomly assign the patient to intervention or control group. This was procedure was performed by a research assistant who was blinded for patients' characteristics.

### Intervention

The intervention consisted of education and self-management of OA symptoms. It was performed by a nurse and aimed to change life style behavior, by improving mobility and physical functioning. On a time-scale the intervention consisted of three parts. Firstly, patients had to prepare for the home visit of the nurse, using an educational leaflet about osteoarthritis (developed by the Dutch College of General Practitioners) and a booklet with health-status charts. The health-status charts were based on the Wonca COOP-charts [17]. The patients needed to fill out their level of exercise, pain-level and their impairments prior to the nurse home visit. The charts were discussed during a 30-minute nurse home visit; this is the second part of the intervention. In this home visit patients got insight in their own OA symptoms. Subsequently, they agreed to try to change one of four life style items (physical exercise, weight loss, use of a walking aid and how to use over the counter (pain) medication). The third part of the intervention was a follow-up phone call after approximately 3 months. In this phone call the nurse evaluated to what extent the patient had been able to adapt his life style change and subsequently what possibly was necessary to maintain this change.

The nurse had undergone a certified education in rheumatology. Patients in the control group received only the educational leaflet about osteoarthritis.

### Outcome measures

Primary outcome measurements were 4 subscales of the Dutch version AIMS2 SF [18] and the Timed Up and Go test (TUG) [19]. The Dutch-AIMS2 SF is an arthritis specific health status scale and we used the following subscales: physical functioning, pain, social functioning and mood symptoms, all scored on a 5-point scale. The AIMS2 SF has been validated for OA in the USA [20] and Germany [21]. The TUG is an objective outcome measure for mobility in older patients: the patient is observed and timed while (s)he rises from a chair, walks 3 meters, turns, walks back, and sits down again. Secondary outcome measures were patient-reported number of contacts with the GP and physiotherapist and whether they used pain medication (over the counter (OTC) or prescribed).

All outcome measures were collected at baseline and after 6 months. Baseline and post-intervention data were obtained in two ways. A patient questionnaire was used to collect all patient reported outcomes. The TUG was performed by the nurse in the intervention group and by a research assistant in the control group for the baseline data. A research assistant measured in all patients the post-intervention TUG, at this stage he was blinded for intervention-control condition.

### Power calculation

To estimate sample size, a power calculation was performed using the subscale lower body limitations of the Dutch AIMS2 SF (Arthritis Impact Measurement Scales Short Form) [18,20] and the Timed Up and Go test [22]. We wanted to detect a small to medium effect (Mean Standardized Difference of 0.4), with alpha 0.05 and beta 0.20. We needed to include 49 patients per group [23]. Anticipating on refusal rates and loss to follow-up we approached 158 patients.

### Analysis

In the analysis, follow-up scores of patients were adjusted for baseline scores [24]. Independent variables were therefore randomization (intervention or control group) and the baseline scores of the respective dependent variables. Data from dropouts and lost to follow-up cases was not available, therefore only cases with data from baseline and after 6 months were included. The analyses were performed using SPSS (version 12) software. Data were checked for normality of residuals. For the primary outcome measure Timed Up and Go test we used a logistic regression technique. TUG times were divided into two clinically relevant groups (=<12 and >12 seconds) on the basis of literature [25]. Dutch AIMS2 SF scales were analyzed with a linear regression technique. The secondary outcome measures (GP visits, physiotherapist visits and use of pain medication) were analyzed using a chi-square test. We did not substitute missing values in any of the scales.

## Results

**Figure 1:**
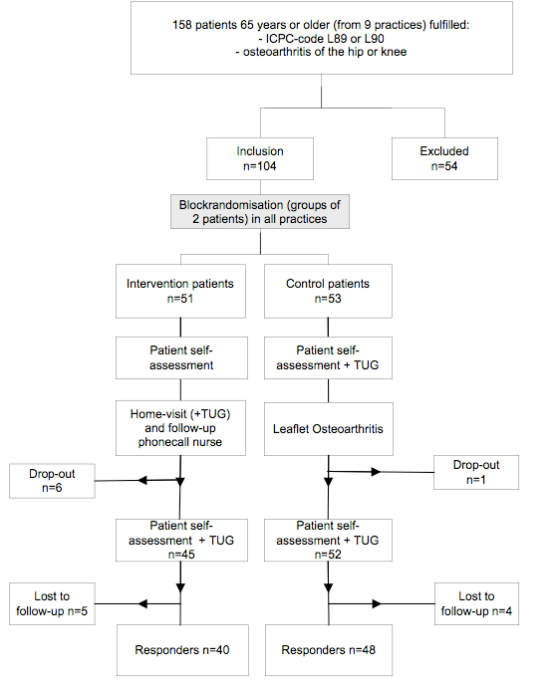


A total of 158 patients were sent an informed consent letter and a questionnaire. After one reminder 125 patients (79.1%) responded. Of these 104 patients were included and randomly assigned (Figure 1). Fifty-one patients were allocated to the intervention group and 53 were allocated to the control group. Fifty-four patients (of the initial 158) could not be included: 33 did not respond to the study invitation, 7 forgot to fill in their names, 12 did not give informed consent, 1 moved to another region and 1 died. Those excluded were not significantly different in age and gender compared to participants. At baseline no differences in self-reported characteristics between intervention and control group patients were detected (Table 1). Due to several reasons seven patients withdrew their participation during the study (motivation problems, moved elsewhere, hip/knee surgery, too severe problems of co-morbidity and treatment by a geriatric specialist) and nine patients did not respond to the final patient self-assessment questionnaire (Figure 1). No differences in self-reported characteristics were found compared to post-intervention responders. Main results are described below and schematically presented in Table 2.

**Table 1:** Patient reported characteristics of included patients at baseline (n = 104)

Characteristic	Intervention N = 51		Control N = 53		T	Chi^2^	p-value
	%	n	%	n			

Gender							
F	76.5	39	75.5	40			
M	23.5	12	24.5	13		0.014	0.91
Type of osteoarthritis							
Knee	52.9	27	54.7	29			
Hip	17.6	9	22.6	12			
Both	29.4	15	22.6	12		0.795	0.67
Education							
Primary or lower secondary	54.0	27	50.0	25			
Upper secondary or further	46.0	23	50.0	25		0.160	0.69
Age		SD		SD			
Mean	75.63	6.68	73.47	6.01	-1.73		0.09
Median	74		73				

**Table 2:** Primary outcome measure (Dutch AIMS-SF)

Outcome measure			Intervention				Control				Comparison^$^
			Pre		Post		Pre		Post		p

		Range	Mean	SD	Mean	SD	Mean	SD	Mean	SD	

AIMS*^#^	Physical	7–35	15.25	4.61	14.56	4.52	14.20	4.40	14.40	4.74	0.36
	Symptoms	3–15	10.10	3.05	8.86	3.34	9.65	3.07	8.87	3.16	0.96
	Social	4–20	11.94	2.70	11.40	2.91	11.43	2.42	11.88	2.76	0.31
	Affect	5–25	12.27	3.35	11.19	3.95	11.23	3.05	11.48	3.64	0.22

* AIMS: The lower the scores, the better the functioning.

^# ^Adjusted for baseline scores.

^$ ^Using linear regression technique

### Primary outcomes

When considering patients' self-reported functioning, intervention patients' mean scores changed towards better functioning. In the control group three out of four subscales in the before-after measurements went in the different direction, thus a worsening in function. However, none of the subscales in the intervention group had a significant improvement compared to the control group (table 2).

With respect to the Timed Up and Go test the shift towards the group 12 seconds in the intervention group was more or less equal to the shift in the control group. One third of the intervention patients (35%) performed the TUG below 12 seconds at baseline and half of the patients after the intervention (50%). For the control group this was 41% and 55% respectively.

### Secondary outcomes

Intervention patients did not visit their GP or physiotherapist more often compared to the control group. In the intervention group 6/40 (15%) patients had 3 or more visits in the past half-year to their GP, compared to 7/48 (14.6%) patients in the control group (p = 0.81). 8/40 (20%) patients in the intervention group received physiotherapist treatment for their osteoarthritis complaints, compared to 6/48 (12.5%) patients in the control group (p = 0.28). Pain medication use did not significantly differ between the two groups (p = 0.49). However, there was an increase in medication use in the intervention group. In the intervention group at baseline 17/40 (42.5%) patients used medication for osteoarthritis pain, whereas post-intervention this was 22/40 (55%) patients. In the control group the numbers were respectively, 24/48 (50%) and 23/48 (47.9%).

## Discussion

This nurse-based intervention did not improve an older OA patient's mobility and functional status, although a non-significant trend towards better functional status was observed. In both study groups patients showed an improvement in functional status. There were no signs of negative side effects, such as more pain among intervention patients, and no signs of increased numbers of visits to the GP or physiotherapist. Numbers were small and only powered to identify a medium difference, and so there is a possibility of a type 2 error. If the trend in effects observed were confirmed in a larger trial, and if such small effects in a common problem such as OA proved to be worthwhile in the long-term, then the intervention might still eventually prove to be effective. However, it is clear from our results that the intervention did not achieve clear or substantial effects. Several considerations for these findings may appear, such as the time between intervention and final measurement may have been to short to detect differences. Also, the intervention itself might have been too simple to detect differences in these outcome measures. However, our results are consistent with a study similar to ours in the same time period [26]. The intervention in this study was slightly more extensive, and their follow-up was 6 months longer; but their findings were that a nurse-led education programme for patients with osteoarthritis (40 years or older) did not benefit these patients. On the other hand, another study showed that a nurse-led intervention aimed at improving non-pharmacologic treatment modalities instead of NSAIDs was effective for OA patients (aged 60 years or older) in primary care [27]. In this study a structured algorithm was used and patients were individually and regularly followed up. A recent trial of self-management of arthritis in patients 50 years and older showed reduced anxiety and improved patients' perceived self efficacy in managing symptoms, but also no significant effects on pain, or physical functioning [28].

The limitations of our study could have interfered with the results. Larger groups, less dropout and longer follow-up might have provided more favorable results. The outcome measures had a number of missing values, despite our efforts to keep the measurements simple and short. The Dutch AIMS2 SF has been validated for rheumatoid arthritis patients, but not for OA patients. However, the US and German version of the AIMS2 SF have been validated for OA patients, with the conclusion of the AIMS2 SF being a reliable and valid instrument to assess the quality of life in primary care patients suffering from OA [21]. The validity and reliability of the TUG might be compromised by the fact that the test was performed at home, on different chairs, and by different observers. At baseline the assessors of TUG times were not blinded for the assignment of subjects to treatment group. There is some evidence that the type of chair does not matter [29], but these factors may have interfered in the validity of the values and may have introduced a bias.

## Conclusion

Non-extensive interventions to improve self-management and life style in OA are, on average, not effective. The counseling may need to be targeted more explicitly to individual problems in order to be successful. Perhaps counseling is only useful for a subgroup of OA patients, such as those with insufficient physical exercise who have a minimum of motivation to increase their physical activities. Furthermore, regular follow up could contribute substantially to the effectiveness of a short intervention. Finally, if a non-extensive intervention is not effective in a patient, more intensive interventions should be available as part of a larger care programme for osteoarthritis. Other health professionals may need to become involved in the delivery of more intensive interventions, such as specialized nurses (rather than generalistic primary care nurses as in this study) and physiotherapists. It is crucial that the effectiveness and feasibility of such interventions and care programmes are tested, before wide-scale implementation is promoted.

## Competing interests

The author(s) declare that they have no competing interests.

## Authors' contributions

RW carried out the study and drafted the manuscript. RG and CvW participated in the concept and design of the study and were important in revising the manuscript. MW participated in the design of the study, helped in coordinating the study and drafting the manuscript. All authors read and approved the final manuscript.

## Pre-publication history

The pre-publication history for this paper can be accessed here:


